# Correlation networks of air particulate matter ($\hbox {PM}_{2.5}$): a comparative study

**DOI:** 10.1007/s41109-021-00373-8

**Published:** 2021-04-23

**Authors:** Dimitrios M. Vlachogiannis, Yanyan Xu, Ling Jin, Marta C. González

**Affiliations:** 1 Energy Technologies Area, Lawrence Berkeley National Laboratory, 1 Cyclotron Road, Berkeley, CA 94720 USA; 2 Department of Civil and Environmental Engineering, University of California at Berkeley, Berkeley, CA 94720 USA; 3 Department of City and Regional Planning, University of California at Berkeley, Berkeley, CA 94720 USA

**Keywords:** Complex networks, Air quality, $\hbox {PM}_{2.5}$ transport, Cross-correlation, Dynamic community detection

## Abstract

Over the last decades, severe haze pollution constitutes a major source of far-reaching environmental and human health problems. The formation, accumulation and diffusion of pollution particles occurs under complex temporal scales and expands throughout a wide spatial coverage. Seeking to understand the transport patterns of haze pollutants in China, we review a proposed framework of time-evolving directed and weighted air quality correlation networks. In this work, we evaluate monitoring stations’ time-series data from China and California, to test the sensitivity of the framework to region size, climate and pollution magnitude across multiple years (2014–2020). We learn that the use of hourly $\hbox {PM}_{2.5}$ concentration data is needed to detect periodicities in the positive and negative correlations of the concentrations. In addition, we show that the standardization of the correlation function method is required to obtain networks with more meaningful links when evaluating the dispersion of a severe haze event at the North China Plain or a wildfire event in California during December 2017. Post COVID-19 outbreak in China, we observe a significant drop in the magnitude of the assigned weights, indicating the improved air quality and the slowed transport of $\hbox {PM}_{2.5}$ due to the lockdown. To identify regions where pollution transport is persistent, we extend the framework, partitioning the dynamic networks and reducing the networks’ complexity through node subsampling. The end result separates the temporal series of $\hbox {PM}_{2.5}$ in set of regions that are similarly affected through the year.

## Introduction

The accelerated industrialization and urbanization of many economically developing countries has led to major air pollution with severe impacts both for human health and the environment (Guan et al. 2014; Huang et al. 2014; Wang et al. 2017). This acute pollution has intensified the frequency of severe haze events which create extremely poor visibility conditions and sharp increase in respiratory diseases (Helble et al. 2000). Such events are accompanied by elevated air quality indices (AQI) and particularly $\hbox {PM}_{2.5}$ levels, which express the density of particulate matter smaller than 2.5 micrometers in the air. Particle matter is either directly emitted into the atmosphere (primary PM) or formed through gas-to-particle conversion (secondary PM), while both types undergo chemical and physical transformations (Seinfeld et al. 1998; Zhang and Cao 2015). Transport of pollutants occurs for various distances depending on meteorological conditions, like wind direction and speed, but also on lifespan, deposition velocities, and altitude of the pollutants (Wang et al. 2017). The significant relations between climate measurements such as temperature and precipitation levels as well as pollution across different areas have been extensively shown in literature proving their non-localized behavior (Hatzopoulou et al. 2017; Gao et al. 2011; HU and YANG 2017; Zhang et al. 2018; Du et al. 2020; Meng et al. 2017). The quantification of those interactions has been evaluated through different measures of similarity, such as cross-correlation (Yamasaki et al. 2008; Ludescher et al. 2013; Fan et al. 2017; Zhang et al. 2018; Du et al. 2020; Zhang et al. 2020; Steinhaeuser et al. 2010; Liu et al. 2018), event synchronization (Boers et al. 2013, 2019) and causal inference (Runge et al. 2015, 2019), and as directed spatial network with correlation magnitudes expressed as network weights.

The complexity in particle matter’s formation, transport and deposition mechanisms makes the design of appropriate pollution regulation policies challenging (Huang et al. 2020). This study aims to gain further understanding of the pathways and timescales of the pollutant dispersal and deposition processes. To that end, we review a recently proposed framework of time-evolving directed and weighted air pollution correlation networks in China. We analyze hourly $\hbox {PM}_{2.5}$ concentration time series, revisiting results in China and contrasting them with California, with available data ranging from January 2014 to May 2020. Incorporating multi-year data and evaluating results from both a small low-magnitude (California) and a large high-magnitude area (China) allows us to comprehensively evaluate and quantify the spatio-temporal dynamics of $\hbox {PM}_{2.5}$ diffusion patterns.

Severe haze events have been consistently occurring in China in large spatio-temporal coverage with $\hbox {PM}_{2.5}$ levels exceeding 100 $\upmu {\hbox {g}}\; {\hbox {m}}^{-3}$ and sometimes reaching even over 800 $\upmu {\hbox {g}}\; {\hbox {m}}^{-3}$ (An et al. 2019). Particularly during wintertime, air pollution aggravates due to excessive energy usage, wind movements (e.g. East Asian winter monsoon), and industrial emissions (Wang et al. 2017), especially in the megacity clusters of the Yangtze River Delta, and the Beijing-Tianjin-Hebei (BTH) regions (Tang et al. 2016; Hagler et al. 2006; Ming et al. 2017; Zhu et al. 2016). As for California, despite the district’s cleaner air compared to China’s heavily polluted atmosphere, relationships between mortality from respiratory causes and long-term exposure to $\hbox {PM}_{2.5}$ have been proven through research studies (Woodruff et al. 2006; Ostro et al. 2007, 2006, 2007). The ambient particle chemistry, size distributions, and temporal patterns of exposure differ from those in other parts of the United States and Canada (Narsto et al. 2004). California’s climate and location lead to intense seasonality in temperature and relative humidity particularly in the inland areas (Hasheminassab et al. 2014). Variability in meteorological conditions has been proven to influence with uncertain sensitivity the formation, accumulation, diffusion and dilution of particle matter (Wang and Ogawa 2015; Aarnio et al. 2002; Tai et al. 2010; Yang et al. 2008). Simultaneously, extreme wildfire events are common during the summer periods in California, affecting $\hbox {PM}_{2.5}$ formation at different extents depending on the distance from wildfires, the altitude of emissions, wind direction, and vertical and horizontal mixing rates (Lee et al. 2016).

The diversity in size, climate and $\hbox {PM}_{2.5}$ magnitude between the two study domains (China and California) allow us to comprehensively evaluate the dynamics of long-range transport and local accumulation of $\hbox {PM}_{2.5}$. In this study, geographical locations comprise monitoring stations in California and cities in China. These are represented as network nodes and weighted links are assigned according to correlation values between pairs of nodes. On a temporal scale, we study the consistency of diffusion patterns across years as well as during the lockdown due to COVID-19 in China, where the levels of anthropogenic emissions decreased. To retrieve comprehensive information from a topological perspective, network partition algorithms are used to determine the underlying organization and randomness of the interconnected nodes (Albert and Barabási 2002; Newman et al. 2011). In the absence of ground truth clusters, aka network communities, modularity maximization has been extensively applied to detect communities (Khanfor et al. 2019; Fazlali et al. 2017; Alzahrani and Horadam 2016; Lancichinetti et al. 2008; Olmos et al. 2020; Kim et al. 2013; Surian et al. 2016) and different approaches for independent matching of communities in dynamic networks have been developed (Hopcroft et al. 2004; Asur et al. 2009; Van Nguyen et al. 2012; Tantipathananandh and Berger-Wolf 2011; Greene et al. 2010; Sun et al. 2015). We apply the method in Ref. Greene et al. (2010) as a means to detect consistently correlated regions from network partitions over yearly data, analyzed at hourly resolution.

In the following sections, we present our data, illustrate our methodology of formulating air pollution networks and compare results in China and California, justifying the link formation method. We then proceed to explore the method’s sensitivity to the lockdown imposed in China to contain the spread of COVID-19. We conclude with dynamic community detection and tracking to identify regions where pollution transport is persistent.

## Data and theoretical background

### Data

We analyze concentration data from outdoor monitoring stations across China and California from Jan. 2014 to May 2020. The California data-set is provided by the U.S. Environmental Protection Agency from 85 regulatory monitoring sites. Because of high instrumentation and maintenance costs, regulatory monitoring is only available at limited locations to examine the compliance to air quality standards (Bi et al. 2020). As for China, the Ministry of Environmental Protection has been releasing the real-time hourly air pollution data since 2013 from monitoring stations across 324 cities. We compare the spatio-temporal dynamics of $\hbox {PM}_{2.5}$ by incorporating multi-year data from both low-magnitude (California) and high-magnitude (China) $\hbox {PM}_{2.5}$ concentration areas.

**Fig. 1:**
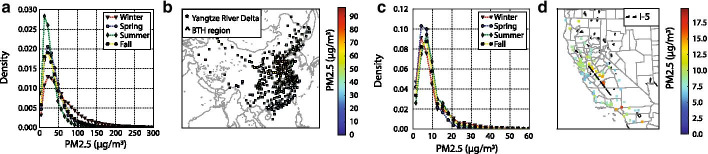
Data Description. **a**, **c** Probability density functions of $\hbox {PM}_{2.5}$ concentrations across seasons. **b**, **d** Spatial distribution of average $\hbox {PM}_{2.5}$ concentrations from Jan. 2014 to May 2020 respectively for China and California

**Fig. 2:**
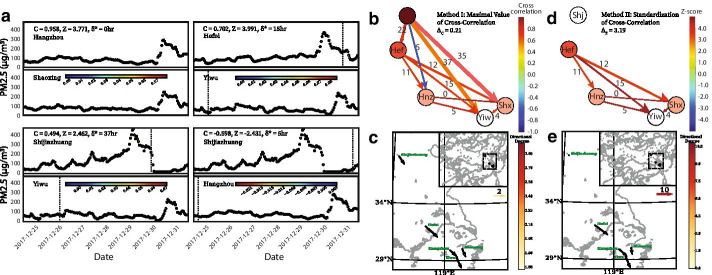
Illustration of network definition methods during haze event in eastern China during December of 2017. **a** Time series of PM2.5 concentrations colored by contribution to the generated correlation among pairs of cities. We showcase the highest positive correlation (Hangzhou–Shaoxing) and Z-score (Hefei–Yiwu) pairs and two pairs, one positive (Shijiazhuang–Yiwu) and one negative (Shijiazhuang–Hangzhou), that generated links only under Method I. **b**, **d** Network visualizations under Methods I and II respectively with nodes colored by out degree, links by weights and width representing the time lag. Method II returns less densely connected networks. In Shijiazhuang, PM accumulates gradually and is more auto-correlated. The generated correlations by Method I are not considered significant in Method II returning Z-scores lower than the critical threshold. **c**, **e** Distribution of directional degrees in the positive correlations networks under Methods I and II respectively illustrating the PM transport spreading southeast

Figure 1 displays the probability density functions of $\hbox {PM}_{2.5}$ concentrations across seasons and spatial distribution of average $\hbox {PM}_{2.5}$ concentrations for both regions. In China, seasonal $\hbox {PM}_{2.5}$ concentrations are significantly higher in the winter and lower in the summer. This pattern is attributed to elevated anthropogenic emissions during winter from fossil fuel combustion and biomass burning for domestic heating as well as cold stagnant weather and temperature inversion favoring pollution dispersion (Zhang and Cao 2015). In contrast, California’s $\hbox {PM}_{2.5}$ distributions do not show an equally strong seasonal dependence. However, higher exceedances of 30 µg/m^3^ still happen during the winter months, primarily due to meteorological reasons and household wood burning. A key contributor to $\hbox {PM}_{2.5}$ accumulation are the wildfires, occurring with high annual frequency and strongly disrupting the atmosphere of the state. The number of wildfires is lower in the winter months than in the annual average (Bay Area Air Quality 2012), explaining the $\hbox {PM}_{2.5}$ peaks during summer months in spite of human generated emissions being down. Overall, in both regions, the seasonal distributions are consistent across all the evaluated years (2014–2020).

As for the spatial distribution in China, Fig. 1b reveals that cities in the north tend to have higher $\hbox {PM}_{2.5}$ concentrations than the cities located in the south, as well as coastal regions’ concentrations are generally lower than in the inland regions. The highest annual average concentration is observed in the Beijing-Tianjin-Hebei (BTH) region which comprises the biggest urbanized megalopolis region in China, including the highest density of coal consumption and heavy industries (Zhang and Cao 2015). To add to the domestic, industrial and agricultural sources, $\hbox {PM}_{2.5}$ pollution is also exacerbated by regional transported contributions from nearby provinces as well as secondary particle formulation. The stagnant climate of the area (weak winds and low boundary layer height) makes it even more susceptible to particle accumulation (Huang et al. 2018). Considering that the size of California is only a fraction of China and that $\hbox {PM}_{2.5}$ is a regional pollutant, the average concentrations within the state are relatively uniform. The majority of sites have an average concentration around 10 µg/m^3^. Similar to China, inland monitoring stations present higher averages, especially those round Interstate Highway 5 (I-5) in the segment connecting the Bay Area with Los Angeles.

### Cross-correlation

Following an analysis framework proposed and applied for the study of environmental complex systems (Yamasaki et al. 2008; Ludescher et al. 2013; Fan et al. 2017; Zhang et al. 2018), we evaluate the dynamics and quantify the spreading and diffusion of $\hbox {PM}_{2.5}$ concentration patterns via correlation networks, where sites are considered as nodes and link weights express the similarity between the time series of sites. The Pearson correlation coefficient is used as a measure of similarity. The cross-correlation function is applied to the fluctuation from average series of length *T* according to:

1 $$\begin{aligned} {\hat{C}}_{i,j}^{(\delta )} = \frac{\langle fX_i(t)\cdot fX_j(t +\delta ) \rangle }{\sqrt{\langle [fX_i(t)]^{2} \rangle }\cdot \sqrt{\langle [fX_j(t+\delta )]^{2} \rangle }}, \end{aligned}$$

where $PM_{2.5}$ concentration on site *i* is denoted by series $X_i(t)$, $fX_i(t) = X_i(t) - \langle X_i \rangle$ is the fluctuation series with respect to average $\langle X_i \rangle = \frac{1}{T} \sum _{t=1}^{T} X_i(t)$, and $\delta \in [-\delta _{max},\delta _{max}]$ represents the timelag. The corresponding time lag is identified as the one returning the maximum of the absolute value of the cross-correlation function $\delta ^*_{i,j} = \hbox {argmax}_{\delta }(|{\hat{C}}_{i,j}^{(\delta )}|)$ and the correlation between sites i and j as ${C}_{i,j} = {\hat{C}}_{i,j}^{(\delta ^*)}$. For $\delta ^* \ne 0$, the correlation is defined as directional, with direction from *i* to *j* for $\delta ^* > 0$ indicating that events in series i precede those of series j.

### Network definition

As in our predecessor studies, the weighted adjacency matrix of the correlation network of week *t*, $W^{C}(t)$ is defined through the maximal absolute value of cross-correlation function (Method I). Links are not assigned to pairs of stations with correlation values below a noise-excluding threshold, $\Delta _C$. The critical threshold is identified by shuffling all the data within the period of evaluation (week *t*), and computed as the average of absolute values of correlations from the permuted data. That way, correlations appearing less in the randomized version do not generate links.

The strength of the correlations can also be quantified by standardizing the correlations $C_{i,j}$ accounting for the significance among all generated ${\hat{C}}_{i,j}^{(\delta )}$ for all the different time lags $\delta \in [-\delta _{max},\delta _{max}]$ by computing the corresponding Z-score (Method II):

2 $$\begin{aligned} Z_{i,j} = \frac{C_{i,j} - \mu _{{\hat{C}}_{i,j}^{(\delta )}} }{\sigma _{{\hat{C}}_{i,j}^{(\delta )}}}, \end{aligned}$$

with $\mu _{{\hat{C}}_{i,j}^{(\delta )}}$ and $\sigma _{{\hat{C}}_{i,j}^{(\delta )}}$ denoting respectively the mean and standard deviation of ${\hat{C}}_{i,j}^{(\delta )}$. As before, the derived weighted adjacency matrix $W^{Z}(t)$ is defined through a critical threshold $\Delta _Z$ which excludes random noise from the Z-scores.

To quantify the influence of a node to its surroundings, we use the weighted directional degree, as introduced in Zhang et al. (2018). For the network with weighted adjacency matrix $W_{i,j}^{C}$, the directional degree is defined as

3 $$\begin{aligned} \vec k^C_i = \sum _{j=1,\delta _{i,j}^{*} > 0}^{N} |W_{ij}^{C}| \vec e_{i,j} + \sum _{j=1,\delta _{i,j}^{*} < 0}^{N} |W_{ij}^{C}| (- \vec e_{i,j} ) \end{aligned}$$

where $\vec e_{i,j}$ the unit vector connecting nodes *i* and *j* defined as

$\vec e_{i,j} = \frac{1}{{\sqrt{{\Delta x}^2 + {\Delta y}^2}}}(\Delta x,\Delta y)$, with $\Delta x$ and $\Delta y$ the latitude and longitude differences between sites *i* and *j* respectively.

### Dynamic community detection

Community detection algorithms aim to decompose a network into sets of sub-units comprising of highly inter-connected nodes (Fortunato and Castellano 2007). A common measure of the quality of the resulted partitions is modularity, which quantifies the difference between the network’s real wiring diagram and a randomly wired diagram as derived under the degree preserving null model. We identify partitions in our networks by applying the Louvain algorithm for faster unfolding of communities, a multilevel technique in which nodes are repeatedly moved to the community of a neighbor if modularity can be improved (Blondel et al. 2008). The results of the Louvain algorithm differ from run to run. In our case, across all of our networks, meaning for every week *t* we are evaluating, the communities generated are very stable. Variations in number of communities are minor and only express a merge or split of communities with not significant jumps of nodes between communities. For each network, the algorithm is being run 100 times. For the minority of nodes that may switch communities across runs, we assign them to the community they appear in the most.

The dynamic nature of our air quality (AQ) networks leads to evolving communities with time. To track the changes in structure, we treat our networks as a time-series of static networks, called timeframes with weekly resolution, according to the method proposed by Greene et al. (2010). We aim to identify the set of dynamic communities $D = \{D_1, \ldots ,D_k\}$ that are present across one or more time steps. Each dynamic community $D_i$ can be represented by a timeline of its constituent step communities, ordered by time, with at most one step community for each step t. The most recent observation in a timeline is referred to as the front $F_i$ of $D_i$. Several studies have focused on detecting the key events that occur in the life cycle of a community (Greene et al. 2010; Asur et al. 2009; Bródka et al. 2013; Dakiche et al. 2019). Those include birth, death, growth, contraction, merging and splitting.

To map the communities detected by the Louvain algorithm at each timeframe to the existing set of dynamic communities *D* we deploy the heuristic threshold-based method introduced by Greene et al. Greene et al. (2010). This method allows for many-to-many mappings between communities across different time steps. To achieve the matching between the community grouping of week *t* with the fronts ${F_1, \ldots ,F_k}$, the Jaccard coefficient for binary sets (Jaccard 1912) is utilized as a measure of similarity. For community *a* of timestep *t*, the coefficient is defined as $J_{(C_{ta},F_i)} = \frac{|C_{ta}\cap F_i|}{|C_{ta}\cup F_i|}$. If the Jaccard coefficient exceeds a matching threshold $\theta \in [0, 1]$, $C_{ta}$ is added to the timeline of $D_i$. If no frontier is matched, a new dynamic community with $C_{ta}$ as first member is created. Between each timestep, after all communities have either been matched to existing dynamic communities or formulated new ones, the fronts of each dynamic community are updated. In our experiments, a range of threshold parameters $\theta \in [0.1, 0.5]$ is investigated. The index is extremely sensitive to small sample sizes and may give erroneous results, especially on data sets with missing observations. Our China dataset consists of 324 different cities (nodes) and we also have instances of missing data from random nodes at certain weeks. Greene et al. Greene et al. (2010) concluded that low values of $\theta$ lead to the most consistent accuracy scores when validating the technique through comparison of the output to the ground truth communities of synthetic networks. Higher values naturally dictate a more conservative matching behaviour, leading to more short-lived communities with lower interpretability interest. Considering the absence of ground truth in our experiment and the volatile spatial behaviour of our communities, a moderately low value of $\theta = 0.2$ is reasonably balancing the trade-off between node assignment accuracy and the identification of the long-lived communities.

### Method illustration

Correlation patterns of AQI have been studied in literature, primarily across sites in China, with time series being grouped and evaluated in seasons (winter, spring, summer, and fall) to account for the seasonal dependence of $\hbox {PM}_{2.5}$ concentration (Zhang et al. 2018; Du et al. 2020; Zhang et al. 2020; Liu et al. 2018). Instead of evaluating correlations over an entire season, we note that a weekly time scale improves interpretability. A season includes multiple haze events occurring on different locations that develop and spread differently, making the causes behind the patterns observed very hard to distinguish. Additionally, transitions from clean to polluted air periods as well as wind circulation in the North China Plain typically exhibit a periodic cycle of 4 to 7 days (An et al. 2019; Guo et al. 2014). Thus to improve the understanding of the underlying dynamical networks, applying the analysis on a weekly basis, makes haze events and extreme phenomena more easily traceable. As for the range of evaluated time lags $[-\delta _{max},\delta _{max}]$, we chose to use $\delta _{max} = 120$ h, as $\hbox {PM}_{2.5}$ life cycle ranges from 3 to 5 days.

In Fig. 2 we showcase the proposed framework for an example network of five cities in northeast China during the last week of 2017. The selection of this particular week is justified by a haze event occurring at the North China Plain and spreading southeast within a couple of days (Huang et al. 2020). In our example, Shijiazhuang, Hefei, Hangzhou, Shaoxing and Yiwu successively experienced at least a day-long of haze pollution. On December 29, Shijiazhuang reached $\hbox {PM}_{2.5}$ concentration levels of over 450 µgm^-3^. Over the next two days, $\hbox {PM}_{2.5}$ peaked at the rest of the cities, all exceeding concentrations of 230 µgm^-3^. The increases were abrupt, causing sharp peaks in the time-series raising the concentrations to four times the average up until that moment during the week. In our example, the majority of generated correlations are positive. Figure 2a showcases the highest positive correlation (Hangzhou-Shaoxing) and Z-score (Hefei-Yiwu) pairs. The contribution of individual pairs of points (timestamps) to the generated correlations among cities are illustrated, demonstrating the significance of haze events and their transport across space and time. We also display two more pairs, one of positive (Shijiazhuang-Yiwu) and one of negative correlation (Shijiazhuang-Hangzhou), that generated links only under the Method I.

Networks are formulated under both methods as defined in the Data and Theoretical Background section. For the method of the maximal value of the cross-correlation function, all links receive absolute values above the critical correlation threshold ($\Delta _C = 0.21$) and therefore are all included in the networks. When accounting for the significance of correlation among all evaluated time lags (Method II), Shijiazhuang is not assigned any links. In Shijiazhuang, PM accumulates gradually (indication of persistence) and is more auto-correlated. The generated correlations by Method I are not considered significant in Method II, returning Z-scores lower than the critical threshold ($\Delta _Z = 3.19$). In Fig. 2b, d the resulting networks are visualized where nodes are colored by out degree, links by weights and width representing the time lag. Method II returns less densely connected networks, as not all links from Method I are considered significant. Figure 2c, e display the spatial distribution of directional degrees in the positive correlations networks. Accounting for the sequence of events as well as for the magnitude of weights, the directional degrees indicate from where $\hbox {PM}_{2.5}$ patterns originate in this example and how they spread southeast. Method II does not detect transport between Shijiazhuang and the rest of the cities due to the low significance of the derived correlation.

## Results

### China

**Fig. 3:**
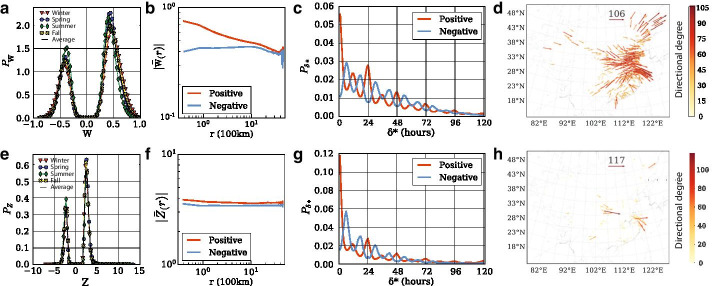
Comparison of Methods in China: the Z-score network definition method formulates less densely connected networks, detecting links from high correlation values achievable only within a short and significant interval of time lags. **a**, **e** Weights display similar bi-modal distributions. **b**, **f** Averages of positive and negative weights at distance r: cities in high proximity are more highly correlated while Z-scores are distance-independent. **c**, **g** Probability distribution function of time lag $\delta$* in positive and negative networks following a characteristic 12 h periodicity. **d**, **h** Spatial distribution of positive directional weighted degree during the last week of 2017: Method II reveals nodes with sharp haze events and their direction of transport

Intercity correlations attain peaks at different time lags. The availability of multi-year data allows us to study the patterns of those correlations and gain better insights on the transport of $\hbox {PM}_{2.5}$. As shown in Fig. 3a the PDF of correlations can be separated into distinct positive and negative parts.

When defining the weekly networks, we exclude the correlation pairs below the critical threshold computed for each particular week. The average correlation among pairs of cities from the perturbed data was calculated at ${\bar{\Delta }}_C = 0.27$. For the positive networks, the decay of the average absolute weights is, consistently across all seasons, following a power law, ${\hat{W}}(r)^{+} = K \cdot r^a$, with exponent $a = -0.166$ and constant $K = 0.718$. The distribution of distances r between positively correlated cities (not shown) is identical with the distribution of the actual distances between cities suggesting that positive correlations appear at random between pairs of cities. Yet, when looking at the magnitude of the correlation, cities closer to each other have higher magnitude of positive weights. In contrast, the average absolute weights in the negative networks have a nearly constant behavior with distance. The fact that negative correlations are distance-independent suggests that their generation is attributed to phenomena like atmospheric circulation which can keep a haze event localized in one node and ventilate another. Whang and Zhang Wang and Zhang (2020) studied the effects of atmospheric circulation on the interannual variation in wintertime $\hbox {PM}_{2.5}$ concentrations over the BTH region in the period of 2013–2018. They identified synoptic circulation types to measure the ability of atmospheric circulation to accumulate, remove and transport air pollutants. The most frequently occurring type is followed by cold, clean and dry air mass transported by surface northwesterly winds, unstable boundary layer and strong horizontal divergence, favoring the improvement in ambient air quality. In contrast, other types characterized by co-occurrence of stable boundary layer, frequent air stagnation, positive water vapor advection and deep near-surface horizontal convergence exacerbate air pollution. The connection of negative correlations in climate and environmental networks with atmospheric waves and oscillations has been highlighted in several studies (Wang et al. 2013; Wallace and Gutzler 1981; Hoskins and Karoly 1981; Aguilera et al. 2020).

Figure 3c demonstrates the characteristic 12 h periodicity that time lags $\delta ^*$ display. Interestingly, positive network time lags peak at 0 h and every 12 h, while the negative network time lags distribution is lagging 6 h. This trend is related to $\hbox {PM}_{2.5}$ daily cycle. The lowest and highest values are reached in the afternoon and night hours respectively according to the daily variation of the boundary layer depth and anthropogenic emissions, with two moderate peaks, one in the morning between 7 am and 10 am, and another in the evening between 7 pm and 10 pm (Zhang and Cao 2015).

Considering how circulation patterns have been shown to affect local air pollution concentration differently, leading to the generation of negative correlations, we deem the positive correlations more suitable to interpret the transport of $\hbox {PM}_{2.5}$ haze events. The directional weighted degrees of the positive correlations network $\vec k^{C^{+}}$ for the sample week used for method illustration are shown in Fig. 3d for the entire China. During the week of Dec. 25th–Dec. 31st, we observe strong weighted directional degrees mostly from east towards the west, showcasing the transport of particles from northwest cities to east and northeast China. The dispersion of $\hbox {PM}_{2.5}$ is a complex phenomenon of multiple species and of different scales highly influenced by meteorological conditions. In China, and especially in the northern part, the climate is regulated by large scale synoptic weather patterns as well as localized circulations. During winter, zonal westerly circulation is common due to occurrence of the East Asian winter monsoon (An et al. 2019) leading to the accumulation and removal in northeastern China.

The Z-score network definition (Method II) formulates less densely connected networks. The Z-scores, which are computed through standardization of the correlation function, share the same shape PDF $P_Z$ as the correlations’ PDF $P_C$ (Fig. 3e), but when calculating the corresponding noise threshold and formulating the weighted adjacency matrix, the derived network is much less connected. The average Z-score among pairs of cities from the perturbed data was calculated at ${\bar{\Delta }}_Z = 3.25$. This method filters out from the networks the links whose correlation might be high but not significant. Pairs of cities which would receive a high absolute correlation value even if the optimal $\delta ^*$ had not been selected are not assigned a link. The average absolute weights in the positive Z-score networks are constant at distance r (Fig. 3f). The more geographically close-by cities do not translate to higher weights in this alternative network definition. Time lags maintain the same periodicity characteristics as in the maximal value of cross-correlation function method.

Based in our analysis with hourly data at a weekly scale and focusing in haze events, we conclude the Z-score approach overcomes the artificial correlations due to persistence and auto-correlations the time-series. These phenomena are frequent in environmental and climate related variables (Guez et al. 2014; Koscielny-Bunde et al. 1998). For the analysis of $\hbox {PM}_{2.5}$, aiming to understand and quantify its spreading, Method II through the standardization of weights depicts a less clear image as how patterns spread. This is seen when comparing Fig. 3d, h. However, it reveals and pinpoints cities and directions where $\hbox {PM}_{2.5}$ pollution transports due to significant events. Such instances are sharp haze events creating peaks in the records that match and return a high correlation value only within a limited interval of $\delta \in [-\delta _{max},\delta _{max}]$ resulting to high Z-scores. In contrast, Method I, accounting for the maximal value of the correlation function, fails to distinguish instances of highly correlated time series under long-range time lags. Therefore, the method is more susceptible to detecting links between nodes due to moderately high similarities of their time series attributed to the low-frequency of $\hbox {PM}_{2.5}$ daily variation.

### California

**Fig. 4:**
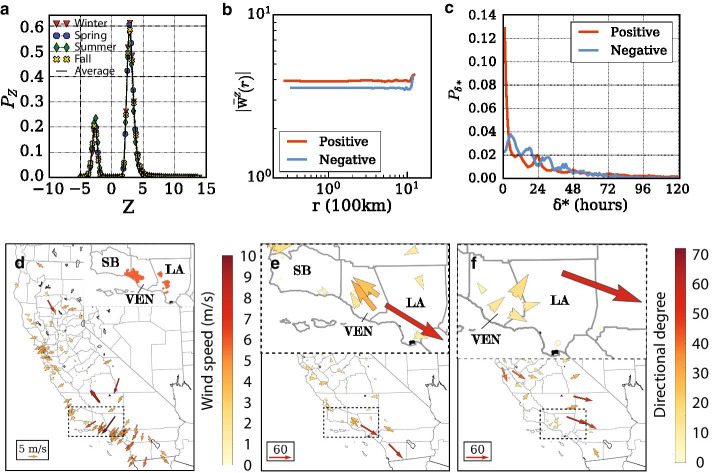
Framework implementation in California, suggesting universality of PM transport patterns. **a** Bi-modal distribution of Z-scores, **b** distance independent Z-scores, **c** 12 h periodicity of time-lags. Wind and $\hbox {PM}_{2.5}$ diffusion during wildfire event. **d** Average daily wind flows on December 7th indicative of the easterly circulation during Santa Ana phase **e**, **f** Spatial distribution of positive directional weighted degrees in the Z networks during (Dec. 4th–Dec. 10th) and after (Dec. 11th–Dec. 17th) the Santa Ana wind stage

In California, $\hbox {PM}_{2.5}$ concentration is more uniform and of lower magnitude due to the region’s smaller size and clearer atmosphere. In general, PM2.5 levels rise and fall within 72 h for all locations throughout the state, with California’s haze events being primarily caused by wildfires which produce time-series with distinctive peaks. For that, the Z-score method is considered more insightful. Overall, we observe a consistency of patterns across both regions. The Z-score distribution (Fig. 4a) displays the same shape as in China. On average the proportion of negative Z-scores in California is 21.31% while in China is 33.49%. The average absolute weights in the Z-score networks remain constant at distance r (Fig. 4b) and the time lag periodicity is sharing the same behavior although not as evident due to the smaller number of available stations in California (Fig. 4c).

We evaluate a wildfire event during December 2017 triggered by the dry and long-lasting offshore Santa Ana winds. Those winds blow out of Southern California’s dry vegetation grown at eastern deserts and mountains (Cao and Fovell 2016). The fires broke out on the week of Dec. 4th, causing traffic disruptions, school closures, hazardous air conditions, and massive power outages. During the same period, the powerful Santa Ana wind event of the season occurred with winds peaking in the mountains surrounding Los Angeles.

Before and going into the Santa Ana phase (Dec. 1st–Dec. 3rd) wind flows were strong northern over the California coast while Southern California’s were weak and of varying directions (Shi et al. 2019). Over the next week (Dec. 4th–Dec. 10th), eastern winds originating from the mountains reached over lower elevation lands (Fig. 4d). Instances of offshore winds exceeding 30 m/s were recorded in the National Climatic Data Center (NCDC). At the same time, wildfires broke out in Thomas (Ventura/Santa Barbara), Creek and Rye (Los Angeles) and Lilac (San Diego). The Thomas fire, affecting mainly the Ventura (VEN) and Santa Barbara (SB) counties (Fig. 4d), grew to 281,893 acres and set the record for California’s largest modern wildfire at the time, leading to significant air pollutant transport. The multiple nuclei of severe haze pollution due to fire induced emissions accompanied by the drastically changing wind flows lead to major turbulence in the weighted directed degrees during that week (Fig. 4e). In the Ventura county, $\hbox {PM}_{2.5}$ was diffused along the coast, with the smoke flume from the central west of the county flowing westward along with the wind, while smoke from the Los Angeles side was pushed further southeast.

Eventually, during the week of Dec. 11th wind flows returned to northern. Transport within the Ventura county weakened while $\hbox {PM}_{2.5}$ from inland Los Angeles was diffusing southeast (Fig. 4f). Overall in the state of California, the strong and highly varying winds both in direction and magnitude help ventilate and spread $\hbox {PM}_{2.5}$, and therefore generating large weights in the Z networks. The weighted directional degrees allow us to evaluate spatial influence of those weights and identify the patterns that are significant for the transport of $\hbox {PM}_{2.5}$ between regions under complex meteorological and environmental conditions.

### Lockdown effect

**Fig. 5:**
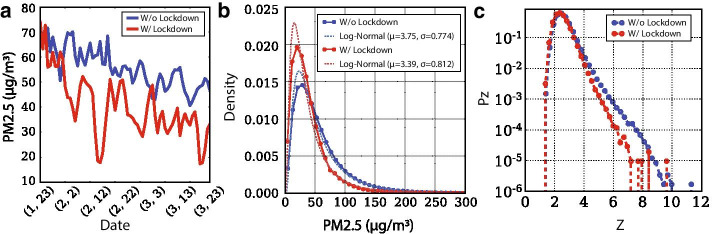
Impact of human mobility drop due to lockdown to pollution transport. **a** Daily $\hbox {PM}_{2.5}$ concentrations. **b** Probability density functions of $\hbox {PM}_{2.5}$ concentrations with and without lockdown. **c** Probability distribution function of Z weights for the with and without lockdown periods: significant links of large weights are less frequently generated under the lockdown suggesting how pollution mitigation can lead to earlier dissipation of haze events

The availability of data until May 2020 allows us to evaluate the sensitivity of our framework to the lockdown imposed due to the outbreak of COVID-19. We are comparing the positive distributions of the Z networks during the lockdown period (Jan 23rd–Mar 28th) of 2020 versus networks from the same dates in 2014 to 2019. This comparison helps us draw conclusions under similar meteorological conditions (same dates across different years) but with significantly reduced emissions from human mobility because the lockdown. Figure 5a depicts the variation of daily $\hbox {PM}_{2.5}$ concentration with and without the implementation of quarantine measures indicating the impact of human mobility to air quality. During that downward trend, the $\hbox {PM}_{2.5}$ distribution followed a log-normal with parameters $\mu = 3.39$ and $\sigma = 0.774$ from a log-normal with $\mu = 3.75$ and $\sigma = 0.812$ during the 2014–2019 years (Fig. 5b). This corresponds to a mean concentration of 39 $\upmu$g/$\mathrm{m}^{3}$ with standard deviation of 371 $\upmu$g/$\mathrm{m}^{3}$, versus 59 $\upmu$g/$\mathrm{m}^{3}$ and standard deviation of 873 $\upmu$g/$\mathrm{m}^{3}$ in the years without lockdown. Formulating the Z-networks during the with and without lockdown periods we observe that the reduction in human mobility led to a drop in the magnitude of the Z weights. This is attributed to the earlier dissipation of haze events due to substantial pollution mitigation. As a result, the diffusion of $\hbox {PM}_{2.5}$ was also mitigated, with significant links of large weights being less frequently generated.

**Fig. 6:**
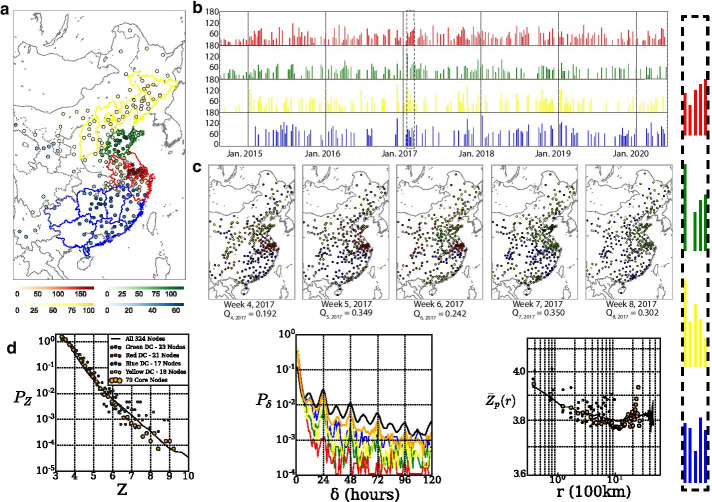
Dynamic Community Detection in China. **a** Spatial mapping of dynamic communities: The four dynamic communities align geographically with megacities and large industrial complexes. **b** Frequency of occurrence and size evolution of dynamic communities: The southeastern community (blue) is the least persistent due to the lower PM concentrations in that region. **c** Methodology illustration during five weeks of 2017, displaying the key events of community evolution tracking. **d** Dynamic communities’ core nodes may sufficiently reproduce the key spatiotemporal patterns of the analysis

### Community detection in dynamic networks

To determine regions with systematic pollution transport behavior, the Louvain algorithm of network community detection was applied to identify partitions in the dynamic AQ networks formulated under the Z-score method. We apply the method in China where more monitoring stations are available and pollution haze events are attributed to less unpredictable and variable events such as the California wildfires. This study emphasizes on long-lived and persistent dynamic communities that appear for several consecutive weeks and persist across our years of evaluation. With $\theta = 0.2$ as the threshold for the Jaccard coefficient, we identify matching communities. Four dynamic communites (DC) prevailed as the most persistent and are displayed in Fig. 6a, with the intensity of the nodes color-coded according to the frequency of occurrence in their matched communities. The DC around the areas of Shanghai and south Jiangsu (red) has its core cities around the Yangtze River including Changzhou, Nanjing and Wuxi. The DC in the province of Shandong (green) contains in high matching frequency Weifang, Zibo and Zaozhuang. The northeastern DC (yellow) spreads along multiple provinces with among others Heibei,Beijing, Tianjin and Liaoning, including as core cities Fushun and Shenyang. As for the southeastern community (blue), the most dominant cities like Zhuzhou, Loudi and Chenzhou are more in-lands with the community similarly spreading over several provinces including Hunan, Jiangxi and Guangdong.

The southeastern community (blue) appears to be the least persistent of the four according to Fig. 6b showcasing the evolution of size for each community. We relate that with the low mean $\hbox {PM}_{2.5}$ concentrations of those regions as shown in Fig. 1. Figure 6c puts on display all of the key events in the Data and Theoretical Background section for the four representative communities. Communities are color-coded in alignment with Fig. 6a and in the case of merged communities the color used corresponds to the community with the highest node participation rate. All four dynamic communities are present on week 4 of 2017 with yellow and green merged together. Next week, the two communities split and only the former remains while the latter disappears. On week 6, the green community reappears exhibiting intermittent behaviour, as it remained unobserved for one timestep. In week 7, green and red fuse together (merging), a state that remains stable during week 8 as well.

We identify the core nodes of the four dynamic communities as the nodes appearing in more than half of the timestamps (weeks) the DC appears. That translates to 21, 23, 17 and 18 nodes for the red, green, blue and yellow dynamic communities respectively. As shown in Fig. 6d, evaluating the networks only using the core nodes is sufficient to reproduce the key temporal and spatial patterns of our analysis. This allows us to reduce the complexity of our networks by sampling 79 out of the 324 nodes of the original data-set. It also offers potentials as a pre-processing method for AQ forecasting as well as informing the re-positioning of stations to monitor areas with no records. As for the intra-community pollution transport patterns, yellow and blue DCs have longer time-lags due to the larger spatial span of their core nodes, as opposed to the more spatially clustered red and green DCs. The absence of longer time-lags within communities is due to the absence of inter-community links.

## Conclusions

Our work has shown that the development of directed and weighted air pollution correlation networks exploiting hourly $\hbox {PM}_{2.5}$ concentration time series with weekly length enables to track haze events and evaluate their diffusion patterns. Shortening the time resolution of the framework provides us with a more interpretable way to understand the dynamics of $\hbox {PM}_{2.5}$ transport. We managed to interpret different type of haze events in both our areas of study, reveal periodicities of pollution transport and relate events to meteorology. In this study, we tested the framework’s sensitivity in China and California. Despite the differences in size, climate and pollution severity, both study regions, share the same probability distribution functions of correlations, with distinct positive and negative parts, allowing us to conclude that the observed patterns are universal. Positive correlations relate to the transport of haze events. Instead, the negatives can be attributed to phenomena like atmospheric circulation and waves synchronizing low magnitude winds at one node allowing for the $\hbox {PM}_{2.5}$ to accumulate and remain localized, as strong winds ventilate the other node. Additionally, time lags maintain the same 12 h periodicity, with the peaks of time lags from positive correlation networks preceding the peaks from negative correlation networks by 6 h. We attribute this behavior to the $\hbox {PM}_{2.5}$ daily cycle, having two moderate peaks, one in the morning between 7 am and 10 am and another in the evening between 7 pm and 10 pm, according to the daily variation of the boundary layer depth and anthropogenic emissions.

We deem that the Z-score method, based on standardization of the correlation function, is more insightful than the maximal value of the correlation function when it comes to analyzing haze events with sharp peaks. It overcomes the artificial correlations due to persistence and auto-correlations within the time-series records. As shown in Fig. 2, it distinguishes significant events generated only by high correlated series in a short, and therefore significant, time range. In that way, it generates less densely connected networks, filtering out links whose correlation might be high but not significant. However, in cases where $\hbox {PM}_{2.5}$ accumulates gradually under stagnant conditions, the maximal value of the correlation method can provide useful interpretations. The framework is implemented to understand the dispersion of a severe haze event at the North China Plain, spreading southeast within a few days. Additionally, we evaluated a wildfire event in California during December 2017, which was combined with the long-lasting offshore Santa Ana winds. In an effort to understand how transportation related emissions affect the $\hbox {PM}_{2.5}$ transport, we observe that the reduction in human mobility due to the imposed lockdown for the limitation of COVID-19 led to a drop in the magnitude of the positive Z weights, supporting the expectation that pollution mitigation can lead to earlier dissipation of haze events.

We extend the Z-score network analysis framework via the Louvain method of community detection in China. Results demonstrated distinct communities presenting consistent air pollution transport patterns and high interconnections between their cities across all years of evaluation. In more detail, four communities prevail as the most persistent grouping the provinces of Shangha–Jiangsu, Shandong, Heibei–Beijing–Tianjin–Liaoning, and Hunan–Jiangxi–Guangdong. The four dynamic communities align geographically with the BTH, Yangtze River Delta, and the Pearl River Delta which correspond to the regions in China with the highest concentrated populations attracted from the river basins and constitute the nuclei of the highest industrial output. Interestingly, identifying the core nodes of the four communities, we showed that they are sufficient to reproduce the key temporal and spatial patterns of the analysis. Apart from significantly reducing the complexity of the original networks, this offers potential as a pre-processing method for AQ forecasting and prediction of extreme pollution events as well as informing the re-positioning of stations to monitor areas with no records. Overall, the network partitioning allows to disentangle spatial patterns, identifying regions where pollution transport is more persistent.

## Data Availability

The California PM_2.5_ dataset is provided by the U.S. Environmental Protection Agency and is available online: https://www.epa.gov/outdoor-air-quality-data. The China dataset is available daily in the national hourly air quality reporting platform run by the China National Environment Protection Agency at: http://113.108.142.147:20035/emcpublish/.

## Abbreviations

PM: Particulate matter
AQI: Air Quality Index
DC: Dynamic community
BTH: Beijing–Tianjin–Hebei
